# The use of orthoses and gait analysis in children with AMC

**DOI:** 10.1007/s11832-015-0691-7

**Published:** 2015-11-04

**Authors:** Åsa Bartonek

**Affiliations:** MotorikLab Q2:07, Astrid Lindgren Children’s Hospital, 17176 Stockholm, Sweden

**Keywords:** Ambulation, Motion analysis, Standing, Heel lift, Knee–ankle–foot orthosis, Carbon spring

## Abstract

**Purpose:**

Arthrogryposis multiplex congenita (AMC) can be described as a complex condition characterized by deformed joints with an intact sensory system. Consequences of muscle weakness and joint contractures in the lower limbs influence walking ability. With orthoses most children achieve functional ambulation. Based on four studies, the aim of this article was to describe gait pattern wearing habitual orthoses, to quantify quiet standing, to test and describe a new orthosis and compare gait differences with regular orthoses, in children with AMC.

**Methods:**

In total 83 children, of which 35 with AMC took part in the studies. All children had underwent clinical examination. Based on joint range of motion and muscle strength they had been prescribed various orthosis types, ranging from insoles to knee–ankle–foot orthoses with locked knee joints. 3D gait and motion analysis was performed during standing and walking with 34 reflective markers aligned with anatomical landmarks.

**Results:**

The findings are presented with respect to each of the included studies.

**Conclusions:**

According to the positive subjective impressions from parents and children, the clinical experiences of our research group, and the objective results from the gait assessments, continuously wearing of orthoses in persons with AMC is recommended.

## Introduction

Arthrogryposis multiplex congenita (AMC) can be described as a complex condition characterized by deformed joints with an intact sensory system; amyoplasia is the most common subtype [1, 2]. AMC has been classified into three groups: disorders with mainly limb involvement (four limbs, lower limbs, or upper limbs), disorders with limb involvement associated with other organ involvement, and disorders with limb involvement and central nervous dysfunction [1].

In children with AMC, the ability to walk depends on the extent of joint range of motion, in particular in the hips and knees as well as in the feet, with the possibility of a plantigrade foot position. Knee joint involvement has been reported in 70 %; most of these are flexion contractures, followed by extension contractures [3]. In children with amyoplasia, hip deformities are common and range from soft-tissue contractures to hip dislocation [3, 4]. Foot deformities are frequently observed, with equinovarus adductus foot being the most common [4, 5].

In children with amyoplasia, muscle weakness, primarily in the hip and knee extensor muscles, influences walking ability [6], and muscle weakness is reported to be more of an influence than contracture severity on walking ability [7]. Equinovarus adductus foot in children with AMC is often associated with plantarflexor muscle weakness [6]. The consequences of calf muscle weakness have been studied extensively in children with myelomeningocele (MMC) with neurological lesions at as low as the sacral and low lumbar levels. Due to the instability in the ankle joint, these children are generally unable to stand still without external support, resulting in increased knee flexion [8]. During standing, body segments should be aligned such that the projection of the center of gravity remains inside the support base. In children with AMC, external shoe wedges have been emphasized to compensate for hip and knee flexion contractures which interfere with static standing. Wedged shoes have also been reported to accommodate plantarflexion deformities and allow the child to bear weight throughout the plantar surface of the foot [9].

Most children with AMC can achieve functional ambulation, and 85 % are reported to be ambulators by the age of 5 years, but a wheelchair may be required for efficient community ambulation [3, 9, 10]. To enhance or facilitate ambulation in children with AMC, orthoses made of different materials and with or without knee-locking mechanisms [11] may be used to compensate for muscle weakness and to support the lower extremities in an aligned position [3, 6, 9]. Due to the essential role of the plantarflexors in walking, orthotic substitutions are essential in patients with plantarflexor paresis. In children with MMC at L4 and L5, ankle–foot orthoses (AFOs) have been shown to improve sagittal plane function by reducing excessive ankle dorsiflexion, increasing plantarflexor moment, and reducing crouch and associated knee extensor moments [12]. One major challenge in the field of orthotics is to compensate for the plantarflexors’ propulsive function; namely, to sustain a plantarflexion moment and to allow simultaneous plantarflexion movement. The posterior leaf spring AFO was tested in cerebral palsy patients with bilateral or unilateral involvement in order to mechanically augment push-off during the stance phase [13]. While it allowed ankle dorsiflexion in midstance, it was not shown to augment ankle function through storage and return of mechanical or spring energy [13]. In children with hemiplegia, orthoses with carbon fiber at the dorsal part of the orthosis were compared with posterior leaf spring AFOs during walking. Results showed a significantly improved third ankle rocker with greater ankle range of motion, angular velocity, and power generation at pre-swing with the use of carbon fiber orthoses [14].

In the past decade, a German orthotics company has been developing an orthosis based on design principles from carbon fiber prosthetic feet. This orthosis was built to accommodate a carbon fiber spring and designed for individuals with plantarflexor weakness due to motor disorders. It was designed to store energy in spring tension during increasing dorsiflexion in midstance and to use this energy at the end of the stance phase for push-off [15]. Results showed more physiological ankle and knee kinematics, implying a functional improvement from the carbon springs compared to classic orthosis, with the patient supported during the complete stance phase [16]. The clinical impression from video observation was that subjects walking with the carbon fiber spring orthosis achieved a more fluent gait, decreased lateral trunk sway, and increased walking velocity compared to conventional orthoses [17].

In our clinical practice, effort is made to analyze the needs of orthoses in children with AMC and to evaluate gait outcome. We also frequently work with shoe adjustments, and we wanted to know how external biomechanical changes influence standing and the alignment of the body segments. This work is based on four studies performed in the motion analysis laboratory in Astrid Lindgren’s Children’s Hospital, Karolinska University Hospital:

- *Study 1*. Eriksson M, Gutierrez-Farewik E, Broström E, Bartonek Å (2010) Gait in children with arthrogryposis multiplex congenita. J Child Orthop 4:21–31 [18]
- *Study 2*. Bartonek Å, Lidbeck C, Brostrom E, Eriksson M, Pettersson R, Gutierrez-Farewik E (2011) Influence of heel lifts during standing in children with motor disorders. Gait Posture 34:426–431 [19]
- *Study 3*. Bartonek Å, Eriksson M, Gutierrez-Farewik L (2007) A new carbon fibre spring orthosis for children with plantarflexor weakness. Gait Posture 25:652–656 [20]
- *Study 4*. Bartonek Å, Eriksson M, Gutierrez-Farewik L (2007) Effects of carbon fibre spring orthoses on gait in ambulatory children with motor disorders and plantarflexor weakness. Dev Med Child Neurol 49:615–620 [21]

The aim of this article was to:

- Describe the gait patterns in children with AMC wearing their habitual orthoses and footwear
- To quantify changes in body segment orientation during quiet standing from heel lifts under the footwear
- To test and describe a new orthosis with a carbon fiber spring that is designed to improve walking by restoring the second and third foot rockers
- To compare gait differences between a carbon fiber spring orthosis and participants’ regular orthoses

All studies were approved by the local ethics committee at the Karolinska University Hospital Ethics Committee, and informed consent was obtained from the participants and their parents.

## Participants

All available children with AMC who were treated in the neuro-orthopedic clinic at Astrid Lindgren’s Children’s Hospital, Karolinska University Hospital, were invited to participate in the studies. All children could adequately understand the test instructions.

### Inclusion criteria for study 1

Independent ambulation with or without orthoses and age between 4 and 18 years. Participants: 15 children with AMC born between the years 1989 and 2003 [eight males, seven females; mean age 12.4 (4.3) years (range 4.7–17.7)]. Six children had four-limb and nine had lower-limb involvement.

### Inclusion criteria for study 2

Children aged 4–18 years with lower limb involvement who were able to stand unassisted for a minimum of 30 s, and had not undergone orthopedic surgery during the previous year nor medical spasticity reduction treatment for the previous 3 months. Participants: 32 children, between March and October 2009, with a mean age of 11.1 (3.7) years, 16 with AMC (nine boys, seven girls) and 16 with cerebral palsy (CP) (12 boys, four girls). Nineteen healthy children, mean age 9.3 (2.1) years (seven boys, 12 girls), constituted the control group.

### Inclusion criteria for studies 3 and 4

All children with neurological motor disorders consecutively included between 2004 and 2007 who were prescribed carbon fiber orthoses by their clinicians for the first time but also had their regular orthoses to hand. Participants: 17 children [six males, 11 females; mean age 11 years 11 months (SD 4 years 5 months); range 3 years 11 months–17 years 4 months). Four children had AMC, 12 children had MMC, and one child had neuropathy with peripheral muscle pareses.

## Methods

### Patient characteristics

All participants underwent a physical examination by the same examiner. The strength of the lower limb muscles was tested manually according to a six-graded scale [22], with grade 0 indicating no muscle strength, grade 1 traces of activity, grade 2 gravity-eliminated movement, grade 3 movement against gravity, and grade 4 indicating movement against gravity with some manual resistance. Grade 5, indicating normal strength, was not given in this study. In the study describing gait pattern (study 1), the children were designated into subgroups with respect to orthosis use and footwear. Group 1, represented by four participants, had reduced knee extensor muscle strength and were grade 3, except for one participant who was grade 4. Group 2, with eight participants, had grade 4 knee extension muscle strength; some of them had insufficient control of knee valgus and knee hyperextension due to the lack of both anterior and posterior cruciate ligaments. Five participants presented a lack of plantarflexors to stabilize the ankle joint. Group 3, with three participants, had sufficient muscle strength to walk without orthoses. One child had a unilateral lift due to leg length discrepancy, and one child had heel wedges to compensate for plantarflexion contractures. In studies 2–4, all participants except for three children had grade 4 knee extensor muscle strength, whereas all had grade 4 hip adductor and hip flexor muscle strength. Muscle strength in hip abduction, knee flexion, plantarflexion, and dorsiflexion was reduced. Joint contractures were defined when measured more than 10° from a neutral position, and plantarflexion contractures as >0° from a neutral joint position. In study 1, two children had hip flexion contractures of 10–20°. Seven children had knee flexion contractures of 10–30°, and eight children (five in group 2 and three in group 3), had knee hyperextension of 10–20°. Seven children had restricted knee flexion of 20–110° from the neutral position. Eight children had plantarflexion contractures of 10–20 °. Fourteen of the 15 participants in study 1 had undergone orthopedic surgery, of which 12 children underwent bony surgery and two children had only soft-tissue surgery.

In study 2, 8/16 children with AMC had ankle contractures of 15–20°, 7/16 had knee contractures of 10–30°, and 2/16 children had hip flexion contractures. The knee extension range was significantly greater in the children who used no orthoses compared to those who used orthoses. In study 3, the carbon fiber spring orthosis was used by two children with lumbar MMC and one child with neuropathy. In study 4, in the AMC children, 6/8 limbs had knee hyperextension of 10° and 4/8 limbs had plantarflexion contractures of 10°. In study 2, one child with AMC had increased muscle tone in the peroneus muscles. Clinical findings and outcomes in children with CP and MMC will not be presented in the current work.

Functional ambulation was assessed according to a five-level scale that has been used previously in children with MMC [23, 24]. In study 1, all participants in group 1 were designated level III, i.e., household ambulators and wheelchair users for long indoor distances. In group 2, one participant was level III and seven participants were level II, i.e., community ambulators who require a wheelchair for long distances outdoors only. In group 3, one participant was level II and two participants were level I, i.e., community ambulators with no need for a wheelchair.

### Orthoses

All orthoses used in the studies were made by the same orthotic company (TeamOlmed, Stockholm, Sweden). Prescriptions of orthoses had been based on the presence of muscle weakness, joint contractures or need for joint stabilization according to the current orthotic programme of Karolinska University hospital. All orthoses were custom-made and individual compensations in orthoses and shoes were made for each participant’s ankle contractures. In studies 1 and 2, the children had been using their orthoses regularly in everyday life. When comparing orthosis types in studies 3 and 4, all participants had been using the new orthosis routinely for 2–3 weeks before gait evaluation and had been instructed to use their former orthoses occasionally during this time. They were tested in both orthoses; first in the orthoses that they wore upon arrival at the gait laboratory. In study 4, a questionnaire was sent out to the parents after the child had used the carbon fiber spring orthosis for longer than 2 months.

**Fig. 1:**
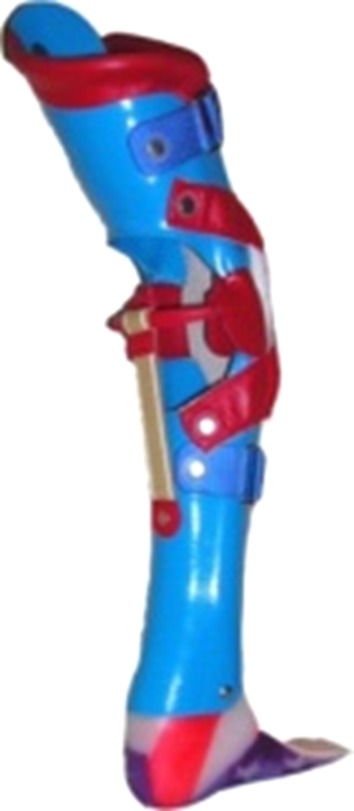
Knee–ankle–foot orthosis with locked knee joints (KAFO-L)
- KAFO-L with a carbon fiber ankle joint (KAFO-L-C) (Fig. 2).

The following orthosis types were used in the studies:

- Knee–ankle–foot orthosis (KAFO) with locked knee joints (KAFO-L) (Fig. 1).

**Fig. 2:**
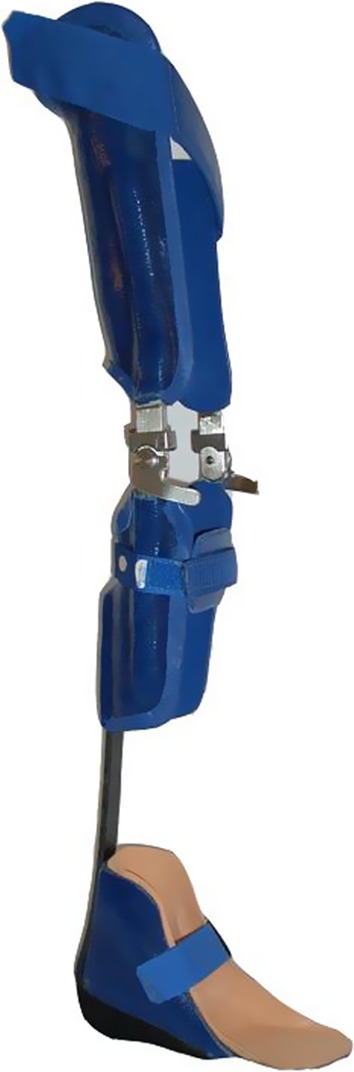
KAFOs with locked knee joints with a carbon fiber ankle joint (KAFO-L-C)
- KAFO with open knee joint and an extension stop as well as a carbon fiber ankle joint (KAFO-O-C) (Fig. 3).

**Fig. 3:**
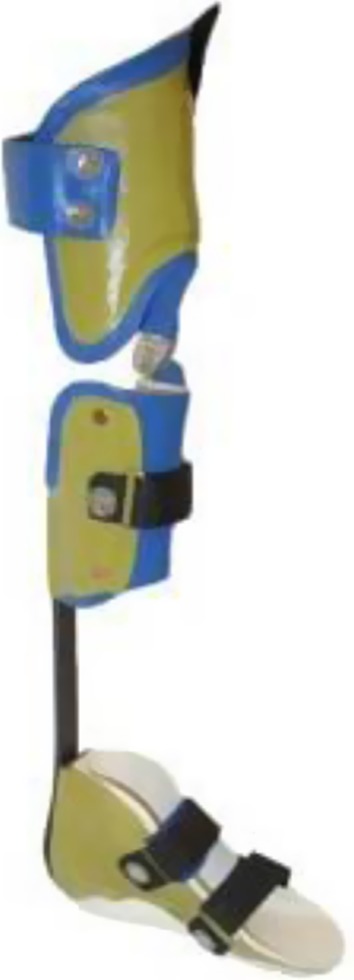
KAFO with open knee joint (KAFO-O) and an extension stop along with a carbon fiber spring ankle joint (KAFO-O-C)
- KAFO with unrestricted sagittal knee motion constructed with over-lap material and a metal screw (KAFO-O) and an over-lap ankle joint connected with a metal screw (Fig. 4).

**Fig. 4:**
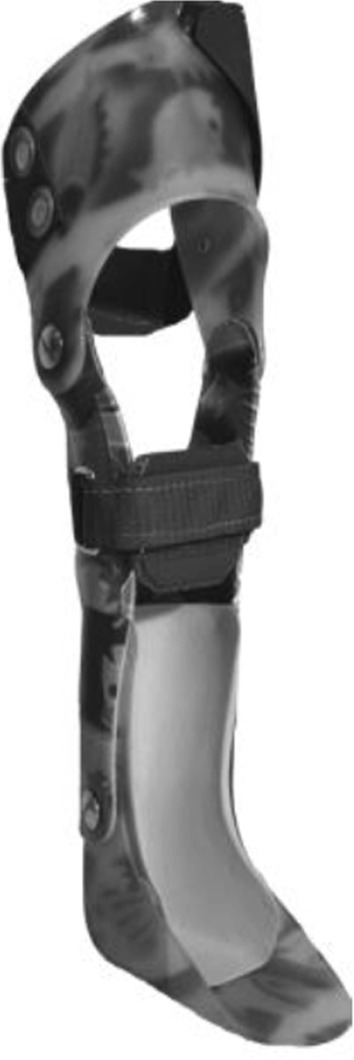
KAFO with unrestricted sagittal knee motion constructed with over-lap thermoplastic material and a metal screw (KAFO-O) and an over-lap ankle joint between a polypropylene foot section and a shank segment of carbon fiber composite material connected with a metal screw
- Ankle–foot orthosis (AFO) with an over-lap ankle joint (AFO-O) connected with a metal screw (Fig. 5).

**Fig. 5:**
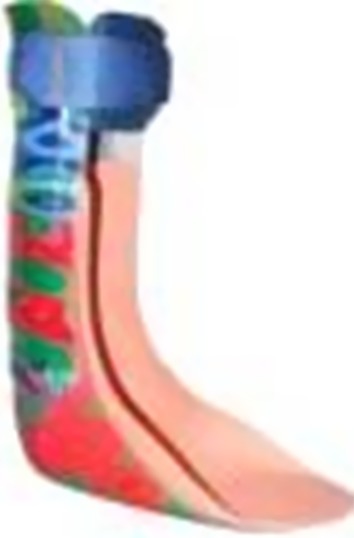
Ankle–foot orthosis with an over-lap ankle joint (AFO-O) between a polypropylene foot section and a shank segment of carbon fiber composite material connected with a metal screw
- Hinged AFO with a dorsiflexion limit and some plantarflexion allowance (AFO-H) Fig. 6).

**Fig. 6:**
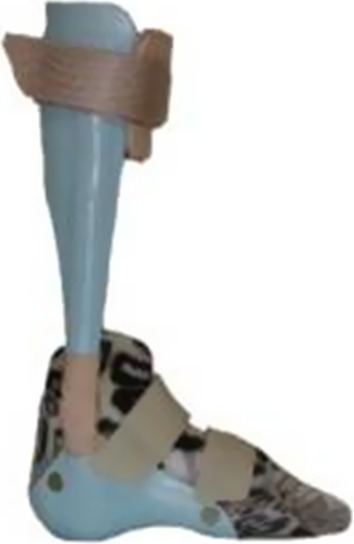
Hinged AFO with a dorsiflexion limit and some plantarflexion allowance (AFO-H)
- Solid AFO with a neutral ankle joint position (AFO-S) (Fig. 7).

**Fig. 7:**
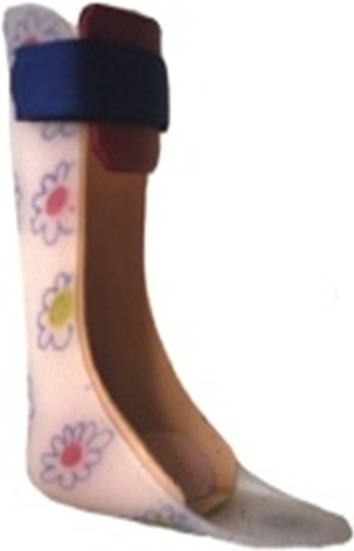
Solid AFO made from thermoplastic material with a neutral joint (AFO-S) (Fig. 7)
- Carbon fiber spring orthosis (CFSO in study 3; SO in study 4) (Fig. 8) with the following characteristics:

**Fig. 8:**
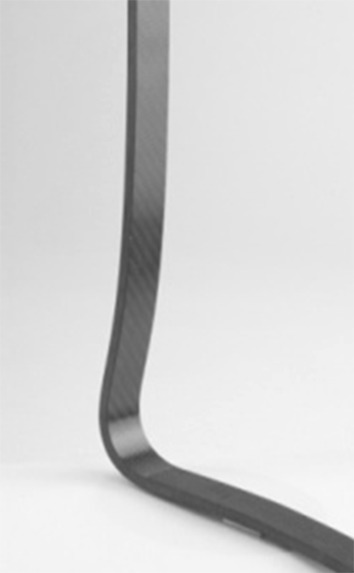
Carbon fiber spring orthosis (CFSO in study 3; SO in study 4) with a polycentric mechanical ankle joint

**Fig. 9:**
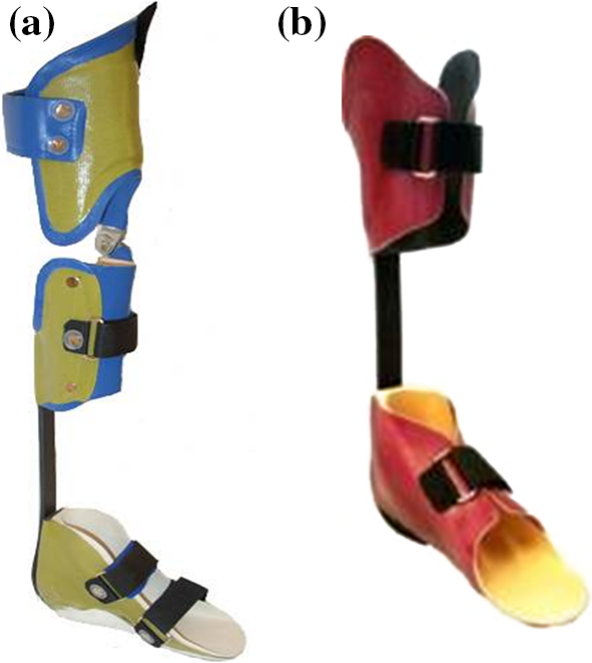
**a** KAFO carbon fiber spring orthosis. **b** AFO carbon fiber spring orthosis (AFO-C)
- Insoles (Fig. 10)

  - Lightweight L-shaped carbon fiber spring component
  - Polycentric mechanical ankle joint
  - Made of a pre-impregnated (“prepreg”) carbon material to enable energy storage during increasing dorsiflexion in mid-stance (that energy is used at the end of the stance phase to aid push-off)
  - Available for individuals 12–90 kg in weight
  - Can be used in AFO or KAFO (Fig. 9a, b)

**Fig. 10:**
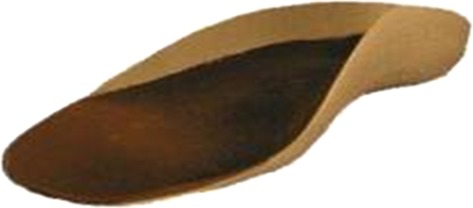
Insoles

The regular orthoses used in studies 3 and 4 were KAFO-O (Fig. 4), AFO-H (Fig. 6), and AFO-S (Fig. 7).

#### Gait and motion analysis

**Fig. 11:**
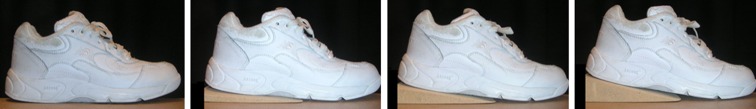
Wedged heel lifts of stiff cork material with heights of 10, 20, and 30 mm and 13.5 mm in length placed bilaterally at the posterior part of the shoe sole and fixed with double-sided tape [19]

The children underwent 3-D motion and gait analysis using an eight-camera motion analysis system (Vicon, Oxford, UK). They were equipped with 34 reflective markers aligned with anatomical landmarks on the head, trunk, and pelvis, and bilaterally on the arms, thighs, shanks, and feet. The lower body was modeled according to the Newington model [25] and the upper body was modeled as the thorax, upper and lower arms, hands, and head according to the plug-in gait model (Vicon). In the children who wore orthoses, the markers were placed as near as possible to the correct anatomical position. The children were asked to walk at a self-selected comfortable pace along a 10-m walkway until complete information from several gait cycles for each side was collected. In study 1, in five of the six children who were able to walk without orthoses, gait analysis was performed barefoot. In studies 3 and 4, walking was tested in two orthosis types, either AFOs or KAFOs corresponding to their regular orthosis model. In study 2, during standing, three different wedged heel lift heights of stiff cork material of 10, 20, and 30 mm and 13.5 mm length (equivalent to mean (SD) 3.7 (1.7)°, 6.4 (2.1)° and 9.8 (2.8)°, were placed bilaterally at the posterior part of the shoe sole and fixed with double-sided tape (Fig. 11). The children were instructed to stand quietly for 20 s. Recording of standing with shoes (“shoe-only condition”) was performed initially, followed by shoes in combination with the three various heel lifts in a randomized order. A video recording was made simultaneously from sagittal and frontal views. All children were tested in their habitual footwear or orthoses.

### Data analysis

In the gait studies, a minimum of three kinematic gait cycles were generated for each subject. The following kinematic parameters were obtained from each gait cycle and averaged for each side to describe gait: range of lateral trunk sway, average trunk tilt, range of trunk rotation, pelvic elevation range, average pelvic tilt, pelvic rotation range, maximum hip abduction, maximum hip flexion and extension, hip rotation at initial contact, knee flexion at initial contact and in mid-stance, maximum knee flexion, knee flexion/extension range, and average foot progression in stance. For group 3 in study 1, the maximum dorsiflexion and plantarflexion were also analyzed. In studies 3 and 4, values for joint moments, powers, and work were also obtained. Time and distance parameters were analyzed, wherein velocity, step length, and stride length were normalized to the leg length. In study 2 (standing), the knee flexion–extension angles were excluded from analysis in four children with AMC who wore KAFOs with locked knee joints. From a frontal view, the children’s more weight-bearing limb was identified through video observation. From a sagittal view, it was checked that the shoe was plantigrade. The angles of each child’s more weight-bearing limb, and with no asymmetry between the limbs during weight-bearing, the right limb, were used for statistical analysis, as for all control children. Among the 20 s recorded, the mean (SD) of 500 continuous representative frames (5 s) during the steady-state standing posture were used for analysis. The heel lift change between shoe-only condition and 10 mm represented an average (SD) angle of 3.4° (1.3°); between shoe-only and 20 mm, this angle was 6.5° (1.6°); and between shoe-only and 30 mm, this angle was 9.6° (2.1°). All statistical analyses were carried out using commercially available software (SPSS version 16.0). Statistical significance was set at *p* < 0.05. Nonparametric statistics were used in all studies.

## Results

### Study 1

Characteristics of the participants in study 1 are shown in Table 1. The children walked with preferred orthoses or footwear. They were assigned to three groups with respect to orthosis use:

**Table 1:** Patient characteristics, limb involvement, functional ambulation, type of orthosis, and patient group (based on orthosis use) in study 1 [18]

Subject	Age (years)	Height (cm)	Weight (kg)	Limb involv.	Func. amb.	Type of orthosis (left/right)	Group
1	12.5	149	31.4	FL	III	KAFO-L-C	1
2	12.7	141	38.1	FL	III	KAFO-L-C	
3	13.0	165	80.0	LL	III	KAFO-L	
4	16.7	165	48.2	LL	III	KAFO-L	
5	4.7	112	19.7	LL	II	Shoe/AFO-C	2
6	6.5	131	27.6	LL	II	KAFO-O-C	
7	7.4	119	21.9	LL	II	AFO-C	
8	9.2	136	28.8	LL	II	AFO-C	
9	12.3	155	37.1	FL	II	AFO-H	
10	15.3	166	44.6	LL	II	AFO-C	
11	16.2	159	46.3	FL	III	KAFO-O-C/AFO-C	
12	17.7	170	55.8	FL	II	AFO-S	
13	12.4	151	37.9	LL	I	Barefoot	3
14	14.0	145	37.1	LL	I	Shoes/heel wedge	
15	13.9	159	38.6	FL	II	Shoes/heel height uni	

*FL* four limbs, *LL* lower limbs, *KAFO* knee–ankle–foot orthosis, *L* locked knee joint, *C* carbon fiber spring ankle joint, *AFO* ankle–foot orthosis, *O* open knee joint with extension stop, *H* hinged,* S* solid

**Fig. 12:**
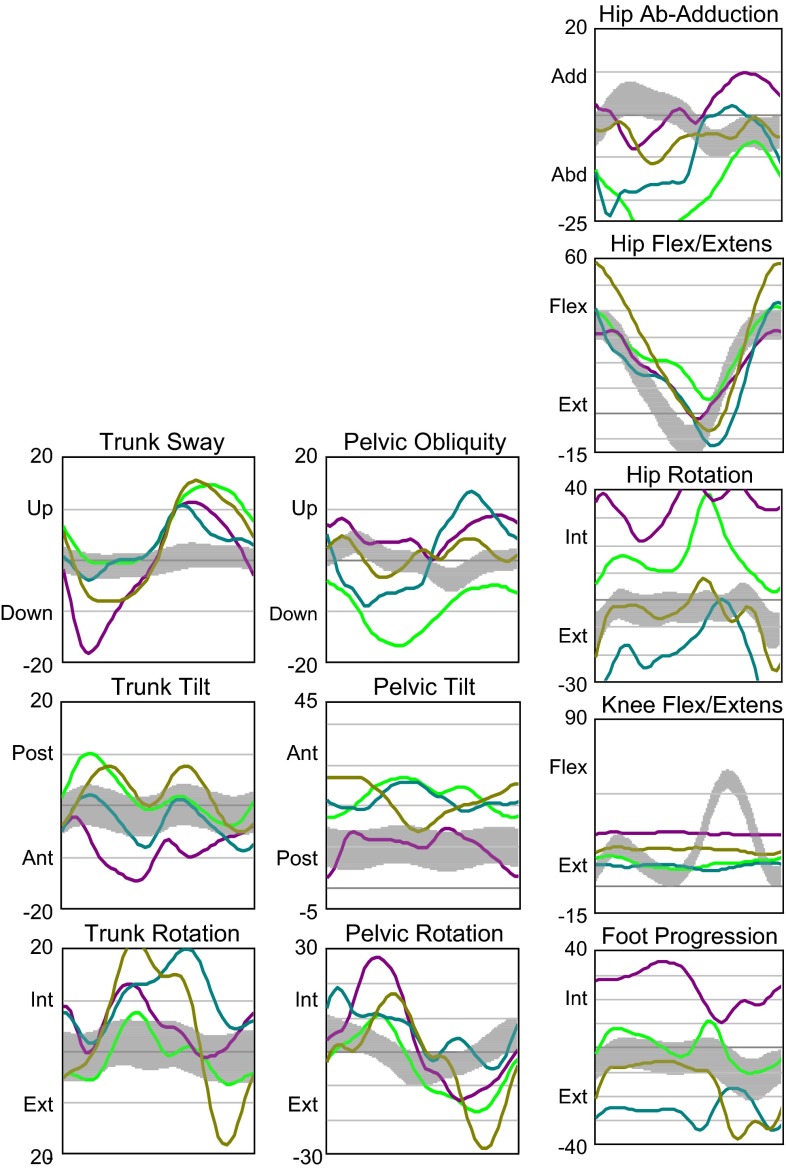
Kinematics group 1: four participants wearing KAFO-L. Each *line* represents one subject, the *gray bar* refers to 23 control children
- *Kinematics group 2* (Fig. 13) (eight participants with knee valgus and knee hyperextension, of which five participants presented lack of plantarflexors to stabilize the ankle joint). The children wore KAFOs and AFOs. Trunk movements were relatively similar in all planes, with slightly pronounced trunk tilt movements towards posterior in stance and swing. There was variation among the participants in pelvic obliquity movements and hip abduction. Maximum hip extension reached the neutral position in almost all children. Knee flexion/extension movements were similar among the participants, and less knee flexion at mid-stance was found compared to group 1. Dorsiflexion and plantarflexion were not analyzed for groups 1 and 2 due to various orthoses and footwear conditions.

- *Kinematics group 1* (Fig. 12) (four participants wearing KAFO-L). Trunk tilt movements tended towards posterior in stance and swing. Pelvis internal rotation was more pronounced in stance, and pelvis external rotation in swing and pelvic lift in swing was observed. Hip abduction during stance was increased, and maximum hip extension almost reached the neutral position. Mean hip rotation at initial contact showed large variations among participants. There was no knee flexion in swing due to the locked orthosis. There was wide variation in foot progression, both internally and externally.

**Fig. 13:**
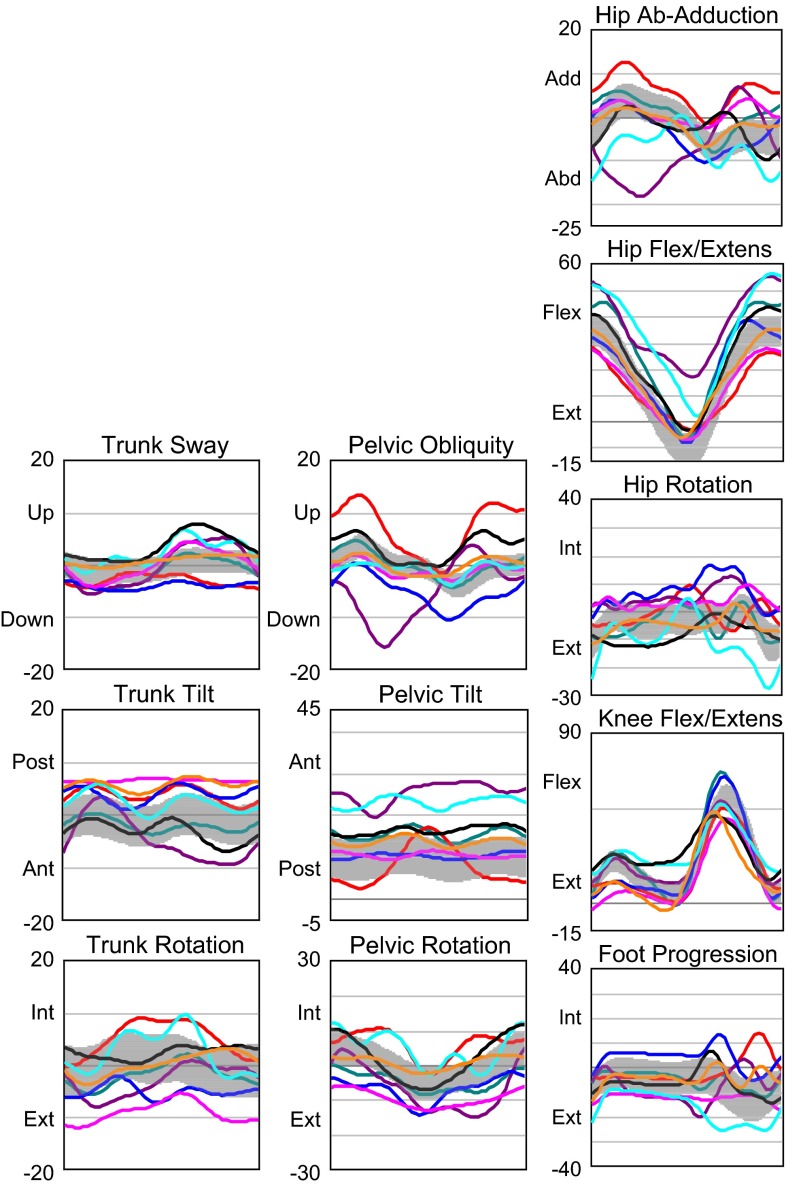
Kinematics group 2: eight participants with valgus and knee hyperextension and five participants presenting lack of plantarflexors. Each *line* represents one subject, the *gray bar* refers to 23 control children
- *Kinematics group 3* (Fig. 14) (three participants with sufficient muscle strength to walk without orthoses, using insoles). In the trunk, the participants were relatively similar in all planes except for one participant who showed greater posterior trunk tilt during the entire gait cycle. The mean anterior/posterior pelvic tilt value was large and neutral hip extension was not reached. Reduced knee flexion during swing was observed in one participant due to restricted knee flexion motion.

**Fig. 14:**
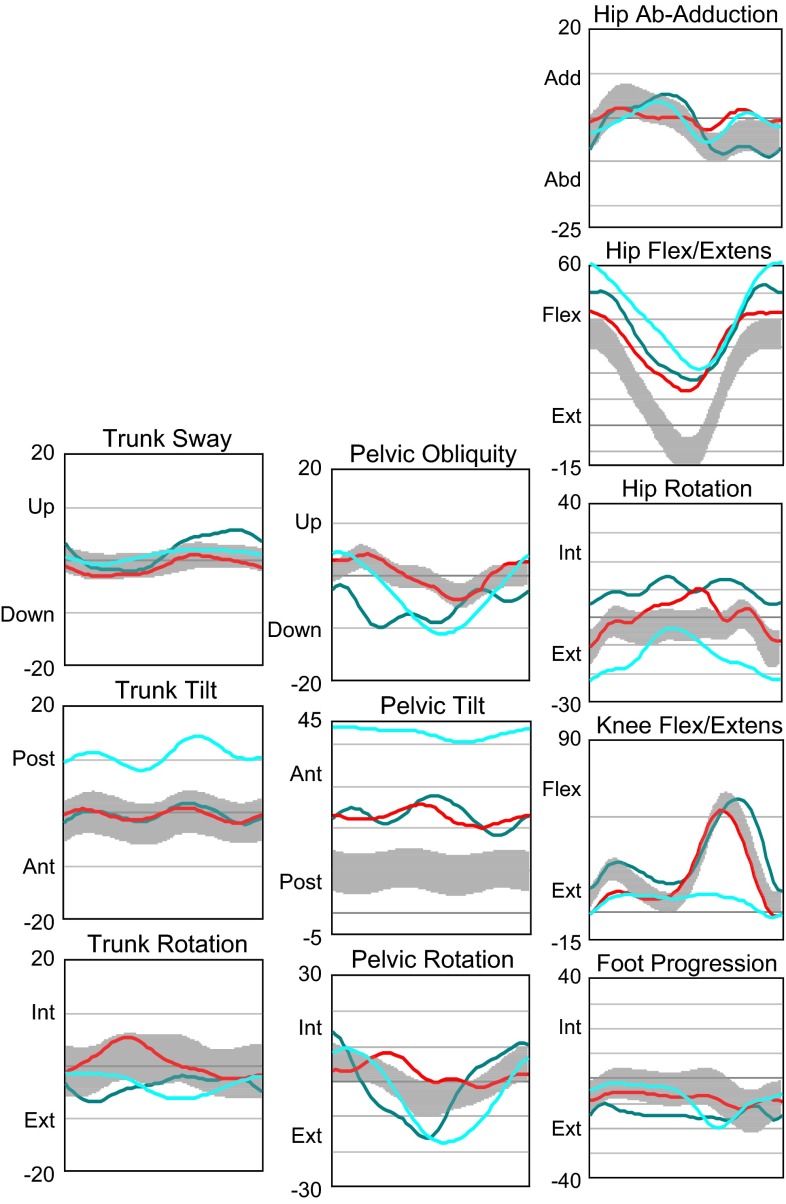
Kinematics group 3: three participants with sufficient muscle strength to walk without orthoses. Each *line* represents one subject, the *gray bar* refers to 23 control children

Trunk lateral sway range, trunk rotation range, and pelvis rotation range all differed significantly among the groups. The maximum knee flexion in swing as well as the mean flexion/extension range were lowest in group 1 as a consequence of the locked orthotic knee joints.

Group 1, as compared to groups 2 and 3 as well as the gait laboratory control group, showed:

- The greatest trunk and pelvis movements in all planes
- The greatest hip abduction
- The lowest cadence and walking speed

Also:

- The maximum hip extension was similar in groups 1 and 2

In addition, group 3 showed:

- The smallest hip extension
- Large deviations from the control data

Cadence and walking speed were significantly lower in group 1 than in groups 2 and 3. Step length and stride length were similar among the groups. Step width was somewhat greater in group 1 than in groups 2 and 3, though the difference was not statistically significant. Step length was similar in the AMC groups to gait laboratory reference values obtained from 23 healthy children aged 5–14 years.

A comparison between walking barefoot and with orthosis was performed in five participants in group 2. With orthoses, the mean cadence was slightly lower than that observed when participants were barefoot. The mean step, stride length, and walking speed increased somewhat with orthoses, but there were no significant differences.

### Study 2

**Fig. 15:**
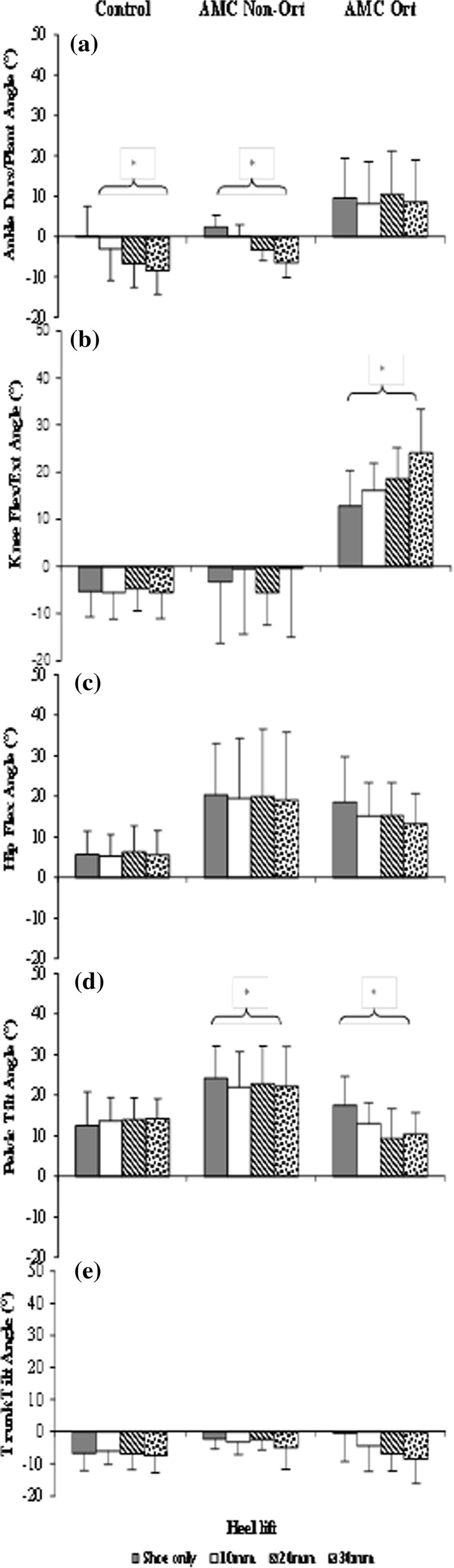
Results for AMC children with orthoses (*Ort*) and no orthoses (*Non-Ort*) who stood for 20 s in shoes only and in shoes with heel lifts 10, 20, and 30 mm high [19]

The results from standing (study 2) for 20 s in shoes only and in shoes with heel lifts 10, 20, and 30 mm high showed differences between the children with orthoses (Ort) and those with no orthoses (Non-Ort). In the Non-Ort group, the ankle angle showed significantly increasingly more plantarflexion with higher heel lifts; in the Ort group, the dorsiflexed position remained unchanged. The knee flexion angle increased significantly in the Ort group with higher heel lifts, whereas the observed hyperextension remained nearly unchanged in the Non-Ort group. In the Ort group, increasing hip extension with increasing heel lifts was observed, but the increase was not significant, whereas hip extension remained unchanged in the Non-Ort group. Particularly in the Ort group, pelvic anterior tilt decreased with increasing heel lift; the corresponding decrease in tilt in the Non-Ort group was not significant. In the Ort group, trunk tilt tended toward posterior with increasing heel lifts. In the control children, the only change observed upon varying heel lifts was significantly increasing plantarflexion (Fig. 15).

### Studies 3 and 4

Results comparing the carbon fiber spring orthosis (Fig. 9) with the childrens’ regular orthoses (Figs. 4, 6 and 7) in study 3 [20] indicated preliminarily improved kinematics, kinetics, and temporospatial parameters as well as positive subjective impressions in three children.

**Fig. 16:**
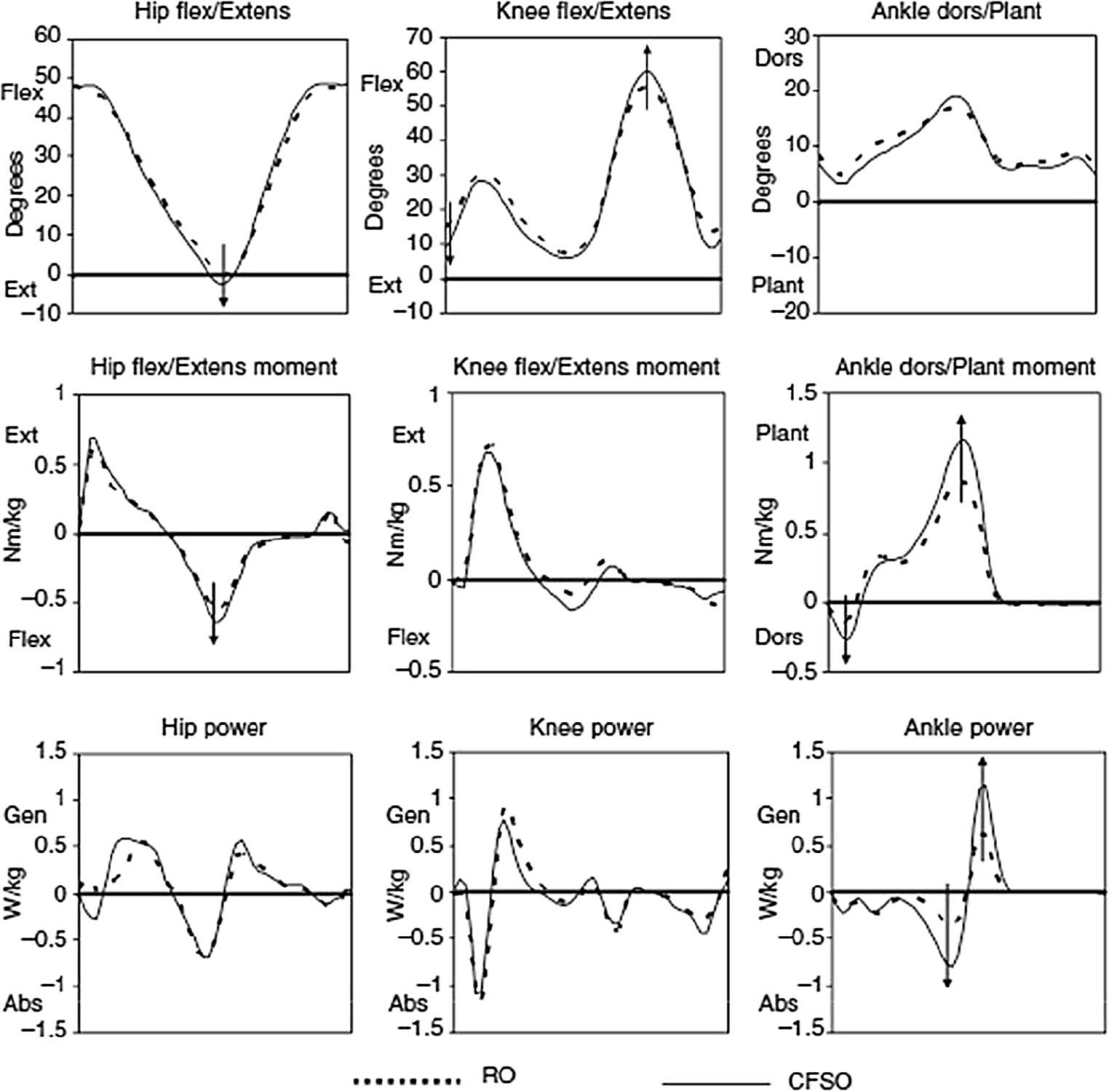
Sagittal plane kinematics (°), joint moments (Nm/kg), and joint powers (W/kg) in the hip, knee, and ankle with the regular orthosis (*RO*) and the carbon fiber spring orthosis (*CFSO*) in the entire group of participants. The mean trace for all of the participants’ right limbs is shown in this figure with *arrows* that illustrate differences found in the entire group. *Flex* flexion, *extens* extension, *plant* plantarflexion, *dors* dorsiflexion, *gen* generation, *abs* absorption [21]

In the larger study (study 4) [21] (Fig. 16), where we examined 17 children who had their regular orthoses available, the carbon fiber orthoses were found to enhance gait function in most participants by significantly improving ankle plantarflexion moment, ankle power generation, ankle positive work, stride length, and walking speed.

With the carbon fiber spring orthosis, trunks were slightly more posterior in the entire group when compared with the regular orthoses, hips were more extended in terminal stance, and knees were more extended at initial contact and more flexed during swing. Stride length and walking speed also increased in the entire group. Parents of 16/17 children answered a questionnaire; all parents reported that their child’s gait had improved and parents of 13/16 children reported that walking velocity was faster (the parents of the other 3/16 children perceived no difference in walking velocity).

## Discussion and conclusion

Among the children who participated in these studies, those in group 1 (study 1) required orthoses to achieve walking ability. They were able to lock and unlock the knee joints independently, as well as to change their position from sitting to standing. All of them used their orthoses throughout the day, which indicates good acceptance of the orthoses. Their gait pattern was characterized by wider steps and more abducted hip movements compared to the other two groups, which is interpreted as increasing their support base to improve balance but also compensating for the extended knees. Further findings from the 3D gait analysis showed differences between the groups in lateral trunk sway, pelvis rotation, and knee flexion/extension range, as well as in cadence and walking speed. Five participants performed gait analysis both barefoot and with orthoses. Time and distance parameters improved during walking with orthoses; however, no significant differences were found based on such a small number of participants. The step length during walking was similar in all groups and to the gait laboratory reference value, which may be attributable to good hip extension strength in all participants, even in those who used orthoses with a locked knee joint. In the standing study (study 2), those who did not use orthoses reacted similarly with increasing heel lifts to the control children in ankle and knee flexion, whereas the orthosis users showed increasingly greater knee flexion. More remarkably, those who did not use orthoses remained in knee hyperextension regardless of heel height, whereas the orthosis users showed a flexed knee position even in the shoe-only condition. The significantly decreasing anterior pelvis tilt and tendency for an increasing posteriorly shifted trunk may raise the risk of increased knee extension moments.

It is therefore a challenge to apply heel lifts that are suited to a particular individual’s conditions taking into account the biomechanical alignment and the orientations of all body segments. This requires the stabilization of the ankle joint, which is the main function of the plantarflexors. The carbon fiber spring orthosis is prescribed not only to fulfill this stabilization function but also to store power and return energy during pre-swing. Preliminary findings suggested that the tested subjects with plantarflexor weakness benefit from the carbon fiber spring orthosis. A larger study could confirm that kinematics, kinetics, and temporospatial parameters improve in terms of the control of tibial advancement, plantarflexor moment produced, ankle power, and work supplement, and in longer strides in almost all participants. Positive work done at the ankle increased in all participants, but it was still lower than the value from the reference database of control children from the same gait laboratory.

Orthotic management with corrective splinting during growth has been recommended in the literature to enable independence [11]. According to the positive subjective impressions from parents and children, the clinical experiences of our research group, and objective results from gait assessments, continuous wearing of orthoses by persons with AMC is recommended.
